# The effect of low intensity shockwave treatment (Li-SWT) on human myoblasts and mouse skeletal muscle

**DOI:** 10.1186/s12891-017-1879-4

**Published:** 2017-12-29

**Authors:** Lise K. Hansen, Henrik D. Schrøder, Lars Lund, Karthikeyan Rajagopal, Vrisha Maduri, Jeeva Sellathurai

**Affiliations:** 1 Department of Clinical Pathology, SDU Muscle Research Cluster (SMRC), Odense University Hospital, Odense, Denmark; 2 Institute of Clinical Research, Faculty of Health Science, University of Southern Denmark, Odense, Denmark; 3 Department of Urology, Odense University Hospital, Odense, Denmark; 4 Paediatric Orthopaedic Unit and Center for Stem Cell Research, Christian Medical Centre, Vellore, India

**Keywords:** Li-SWT, Skeletal muscle regeneration, Vascularization, Angiogenesis, Myoblasts

## Abstract

**Background:**

Transplanting myogenic cells and scaffolds for tissue engineering in skeletal muscle have shown inconsistent results. One of the limiting factors is neovascularization at the recipient site. Low intensity shockwave therapy (Li-SWT) has been linked to increased tissue regeneration and vascularization, both integral to survival and integration of transplanted cells. This study was conducted to demonstrate the response of myoblasts and skeletal muscle to Li-SWT.

**Method:**

Primary isolated human myoblasts and explants were treated with low intensity shockwaves and subsequently cell viability, proliferation and differentiation were tested. Cardiotoxin induced injury was created in *tibialis anterior* muscles of 28 mice, and two days later, the lesions were treated with 500 impulses of Li-SWT on one of the legs. The treatment was repeated every third day of the period and ended on day 14 after cardiotoxin injection.. The animals were followed up and documented up to 21 days after cardiotoxin injury.

**Results:**

Li-SWT had no significant effect on cell death, proliferation, differentiation and migration, the explants however showed decreased adhesion. In the animal experiments, qPCR studies revealed a significantly increased expression of apoptotic, angiogenic and myogenic genes; expression of *Bax, Bcl2*, *Casp3*, *eNOS*, *Pax7, Myf5* and *Met* was increased in the early phase of regeneration in the Li-SWT treated hind limbs. Furthermore, a late accumulative angiogenic effect was demonstrated in the Li-SWT treated limbs by a significantly increased expression of *Angpt1, eNOS, iNOS, Vegfa*, and *Pecam1.*

**Conclusion:**

Treatment was associated with an early upregulation in expression of selected apoptotic, pro-inflammatory, angiogenic and satellite cell activating genes after muscle injury. It also showed a late incremental effect on expression of pro-angiogenic genes. However, we found no changes in the number of PAX7 positive cells or blood vessel density in Li-SWT treated and control muscle. Furthermore, Li-SWT in the selected doses did not decrease survival, proliferation or differentiation of myoblasts in vitro.

**Electronic supplementary material:**

The online version of this article (10.1186/s12891-017-1879-4) contains supplementary material, which is available to authorized users.

## Background

Satellite cell (Sc) derived myoblasts are widely used in regenerative medicine for engineering skeletal muscle tissue. Their successful transplantation, however, has been hampered by poor survival and poor engraftment in transplanted tissue [1–3]. Newer scaffolds have improved the success rates of myoblast transplantation [4, 5], but insufficient vascularization is still a limiting factor [6]. Common methods for improving vascularization include employment of gene-modified cells expressing angiogenic factors and scaffolds releasing angiogenic factors like VEGF and IGF1 [7, 8].

A promising, non-invasive, indirect technique to improve vascularisation is a low intensity shockwave treatment (Li-SWT). This is increasingly employed in regenerative medicine and wound healing [9–11] although clinical studies conducted so far have not advocated the use of SWT in the clinic. More research is needed to establish the clinical effect of SWT from a clinical perspective. Shockwaves are transient high-pressure acoustic pulses that can be generated by different mechanical principles [12]. Shockwaves affect tissue by a direct mechanical force and by creating bubble cavitations that burst, generating a jet flow affecting nearby cells [12–14]. This results in minor intra- and extracellular damages, shear stress and conversion of mechanical force into chemical activity (known as mechanotransduction) [14, 15] inducing a regenerative response in the involved tissue.

In vitro studies with Li-SWT have shown to enhance certain functions involved in the behavior of specific cell types. Li-SWT increases proliferation, migration and secretion of collagenase in tenocytes treated with 1000 impulses of 0.14 mJ/mm^2^ [16, 17]. Human osteoblasts treated with Li-SWT (500 impulses, 0.06 and 0.5 mJ/mm^2^) show a dose-dependent increase in proliferation and increased expression of the genes *PTHLP* and *PTGER3* which are involved in bone development and osteoblast differentiation [18]. Li-SWT (300 impulses, 0.1 mJ/mm^2^) treated mouse endothelial progenitor cells showed increased expression of angiogenic cytokines (specifically *Vegf, Nos3, Angpt1, Angpt2)* and *Kdr (a gene that codes for the protein vascular endothelial growth factor receptor 2*) [19]. Primitive human cardiac cells showed increased KDR protein levels when treated with Li-SWT (800 impulses, 0.1 mJ/mm^2^) [20].

In vivo studies with Li-SWT have shown improved tissue regeneration, greater vascularization and altered immune responses. Studies in rodents have shown increased perfusion, survival, and vessel density in skin flaps in addition to increased gene expression of *eNOS* and *Vegfa* [21–23], and increased perfusion of ischaemic adductor muscle [24] after Li-SWT ranging from 200 to 750 impulses, (0.1 mJ/mm^2^). Altered immune response was seen in cremaster muscle tissue as decreased rolling and transmigration of leukocytes through the endothelium as well as down regulation of *iNOS* gene after Li-SWT (500 impulses 0.1 mJ/mm^2^) [25, 26].

Li-SWT is a widely used treatment of tendon and bone-related conditions such as tendinopathy, plantar fasciitis and non-union of bone [27]. In addition, much ongoing research is focused on the ability of Li-SWT to ameliorate muscle pain, spasticity and peripheral arterial disease [28–30]. However, there are no published results in the indexed literature on the effect of Li-SWT on myoblast and skeletal muscle regeneration after acute injury. Thus, the question whether Li-SWT can boost regeneration and improve vascularisation of the muscle has not yet been addressed.

### Study objective

The objective of this study is to address the effects of Li-SWT on regenerating skeletal muscle. For this purpose we have conducted following studies:

1. An in vitro study on human myoblasts was conducted to look selectively at the effects of Li-SWT on myogenic stem cells.
2. An in vivo study was conducted in mice. The animals received an acute myotoxic injury on tibialis anterior muscle, followed by treatment with Li-SWT to study the effect of Li-SWT on muscle regeneration.

## Methods

### Shockwave application

A handheld Duolith SD1 equipment (Storz, Tägerwilen, Switzerland) was set at the intensity 0.10 mJ/mm^2^, 5 Hz (5 impulses pr. second), with 300, 500, 1000, or 1500 impulses given continuously in one treatment lasting 60s, 100 s, 200 s, or 300 s respectively. The treated area was covered with EKO GEL (ultrasound transmission gel, Ekkomarine Medico A/S, Denmark) and the focused shockwaves were delivered with a focal area (penetration depth) at 0-30 mm.

For the in-vitro experiments, a cryotube (1.8 ml) filled with Ultroser G (UG) medium containing 2.5 × 10^5^ cells and a cryotube filled with growth medium (GM which consisted of DMEM with 10% FBS and 1% PSA (Penicillin-Streptomycin-amphotericin B, Life Technologies)) containing pieces of muscle explants of approx. 0.5 cm^3^. These were covered with EKO GEL and treated once with 300, 500, 1000 or 1500 impulses of Li-SWT immediately before culturing.

The control cells were transferred to cryotubes at the same time as the samples treated with Li-SWT before culturing, but did not receive shockwave treatment.

### Myoblast isolation and culturing

Human myoblasts were isolated from biopsies taken from the *vastus lateralis* muscle of young men (18–20 years). These samples were obtained from a previous study [31]. Biopsies free of connective tissue were minced and digested with 0.3% collagenase type II (Medinova Scientific) for 40 min. in 37 °C water bath. The suspension was titrated with a 1 ml pipette, cold HBSS (Hanks Balanced Salt Solution) with 10% FBS was added and the suspension was pelleted and re-suspended in 37 °C HBSS with 10% fetal bovine serum (FBS) and filtrated first through a 100 μm then through a 40 μm Falcon Cell Strainer. The isolated satellite cells were cultured in GM and plated on extracellular matrix (ECM, Sigma-Aldrich, MO, USA) coated dishes (Nunclon, Nunc). During every passage, the number of fibroblasts was reduced by pre-plating the cells for 20 min. at 37 °C on untreated NUNC dishes. The non-adherent cells were harvested, expanded and aliquots were frozen and kept in liquid nitrogen.

For the in vitro experiments the cells were thawed and seeded on ECM (Extracellular Matrix, Sigma-Aldrich, MO, USA) coated flasks and coverslips (Fischer Scientific, MA, USA) and cultured in growth medium (DMEM w. hepes and DMEM w. glutamat (Life Technologies, CA, USA)) with 2% Ultroser G (Pall), 2% FBS (Fetal bovine serum, Life Technologies) and 1% PSA (Penicillin-Streptomycin-amphotericin B, Life Technologies).

### Explant culture conditions

Human explants were obtained from the *m.quardriceps femoris* and *m. soleus* (females aged 78 and 31), and cultured in a growth medium with 10% FBS and 1% PSA. The media was changed 9 days after Li-SWT and afterwards twice a week and the explants were harvested after 17 days.

### Myoblast viability, proliferation and differentiation after Li-SWT

Myoblast survival was measured by tryphan blue staining (Sigma-Aldrich) immediately after shockwave treatment. In order to examine the proliferation rate after Li-SWT, myoblasts were cultured in 10 mM BrdU in UG media (cumulative BrdU incorporation), and coverslips were harvested at days 1, 2, 3 and 4 after treatment. The differentiation ability of myoblasts after Li-SWT was tested by culturing them in a differentiation medium (DMEM with 2% FBS, 1% PSA and 25 pM insulin (Actrapid from Novo Nordisk, DK)) and cells were harvested on days 2, 4, and 6 after Li-SWT.

### Animal experiment

Twenty-eight female mice aged 10 weeks (C57BL/6NTac) received a single lesion in both of their tibialis anterior muscle (TA) through an injection of 50 μl cardiotoxin, CTX (10 μM). Two days later the mice were treated with Li-SWT for 100 s (500 impulses of 0.1 mJ/ mm^2^ (5 Hz), 1.635 joule) on the left leg, while the right leg functioned as control. Prior to the shockwave application, the skin of the leg was depilated to eliminate shockwave interferences. The treatment was repeated every third day of the period, ending on day 14 after CTX injection. Four mice were euthanized at each time point on days 2 (5 h after first Li-SWT treatment), 3, 5, 7, 10, 14 and 21 after cardiotoxin injection.

The TA muscles were removed from both legs and cut into halves. One half was stored in RNAlater (Ambion, Life technologies) in −20 C° for qPCR analysis. The other half was fixed in formalin for 24 h and embedded in paraffin for histological studies.

### RT-qPCR

#### Cell cultures

Gene expression studies were performed with 3 biological replicates.

In order to examine gene expression under proliferation, cell cultures were harvested 5 h, 12 h, 24 h, 48 h, 72 h and 96 h after Li-SWT and lysed with 0.5–1 ml trizol (Life Technologies, CA, USA).

#### Animal experiments

Of the four mice euthanized, three were used for RT-qPCR. The muscles were lysed in 1 ml trizol using MagNA lyser beads and processed on Magna Lyser Instrument (Roche Applied science, DK) for 2 × 20 s at 6500 rpm, following this RNA was extracted.

cDNA was synthesized from 500 ng RNA using High Capacity cDNA Reverse Transcription kit (Life Technologies) and qPCR performed on Quantstudie 12 K flex (Life Technologies) using custom designed 384 well TaqMan Array Micro Fluidic Cards. The data were analysed in qBasePlus (Biogazelle), and quality control was set by excluding replicates with >0.5 deviation from quantification cycle (Cq). Reference genes were selected using Genorm (M–value <1.5). The results for the individual genes are shown as mean fold change to the lowest normalised Cq value, which was set to 1. The following genes were examined in myoblast cultures*: TGFb1, SMAD3, SMAD7, MKI67, CYCLIND1, P21, TP53, CASP3, PAX3, PAX7, MYF5, MYOD1, MYOG, MEF2A, MET, HGF* and *KDR*. *GAPDH* and *TBP* were used as reference genes.

In the animal experiment the genes examined were: *Bax, Bcl2, Casp3, Il1a, Il1b, Il6, Ccl2, Ccr2, Tnf, Pax7, Myf5, Myod1, Myog, Myf6, Mstn, Met, Hgf, Mef2c, Tgfb1, Angpt1, Angpt2, iNOS, eNOS, Vegfa, Kdr, Pecam1, Cd34, Fgf2 and Fgfr1*. *Rn18s, Tbp* and *Tfrc* were used as reference genes. All qPCR data (fold changes) for the in vitro and in vivo experiments can be seen in Additional file 1.

### Immunohistochemistry

#### Cell cultures

For detection of BrdU, samples were fixed in 96% alcohol for 30 min before incubation in 2 N HCL with 0.5% Triton X-100 for 30 min. Afterwards samples were neutralised for 3 × 5 min in NaBH_4_ solution (1 mg/ml), followed by incubation of mouse-anti-BrdU (1:20, Bu20a clone, Dako, DK).

For detection of NCAM, explant samples were fixed in 4% formalin for 5 min followed by incubation in Triton X-100 for 5 min, prior to incubation with mouse-anti-NCAM (1:100 CD56 Leu-19), Becton Dickinson, DK).

For detection of myogenin, samples were fixed 15 min in 4% formalin followed by incubation in 96% ethanol for 10 min. After rinsing in water the samples were incubated in Tris-EGTA buffer at 95 °C for 15 min, followed by incubation with mouse-anti-myogenin (1:800, F5D, Dako). Powervision (Leica Biosystems) was used for detecting BrdU, NCAM and myogenin.

For detection of F-actin, samples were washed twice in preheated (37°) TBS (Tris-buffered saline) and fixed in 4% formalin for 10 min and incubated in 0.1% Triton-X100 for 5 min, before incubation in phalloidin (1:40, Alexa Flour 546 Phalloidin, Lifetechnologies). After washing with PBS, samples were mounted with Vectashield (Vector Lab, UK).

#### In vivo experiment

Staining of muscle paraffin sections was carried out on Dako Autostainer Plus (Dako) using Dako EnVision + kit and the following antibodies: rabbit anti-rat IgG (Dako) followed by rat-anti-Cd45 (1:100, 30-F11, Pharmingen, BD, DK), rabbit-anti-vWF (1:2000, Dakopatts A/S, DK), mouse-anti-myogenin (1:200, clone F5D, Dako) and mouse-anti-Pax7 (1:20, Hybridoma Bank, IA, USA). The sections were blocked for endogen biotin by Avidin/Biotin Blocking kit (Vector Lab, UK) prior to incubation with myogenin and pax7.

### Morphometrics and statistical analyses

CAST software (Visiopharm A/S, Hørsholm, DK) was used for all morphometric analysis. Systematic, random counts were performed on blinded samples including the entire cell containing area. In assessment of BrdU incorporation and cell adhesion 10% of the coverslip area was counted, while 25% was counted when quantifying myofibers (≥ 3 myogenin + nuclei) and 2% when determining the myogenin fraction in cell cultures.

Quantification of the immunostained paraffin sections from the animal experiment was performed by counting MYOG+, PAX7+ and CD45+ cells and vWF+ vessels in 100% of the selected areas (regenerating and non-regenerating part of the TA muscle). As the size of the muscle injury induced by cardiotoxin varied between animals, the selected regenerating area varied, too, why the data is presented as positive events per cm^2^.

Statistics were performed in Graphpad Prism, version 5.0 (Graph Pad Software Inc., LA, USA). All data are shown in mean + SD (standard deviation) and results are tested by paired t-test in order to detect difference between Li-SWT treatment and control. *P*-values below 0.05 were considered significant.

## Results

### In vitro cell survival, proliferation and apoptosis of myoblasts were not affected by Li-SWT

**Fig. 1:**
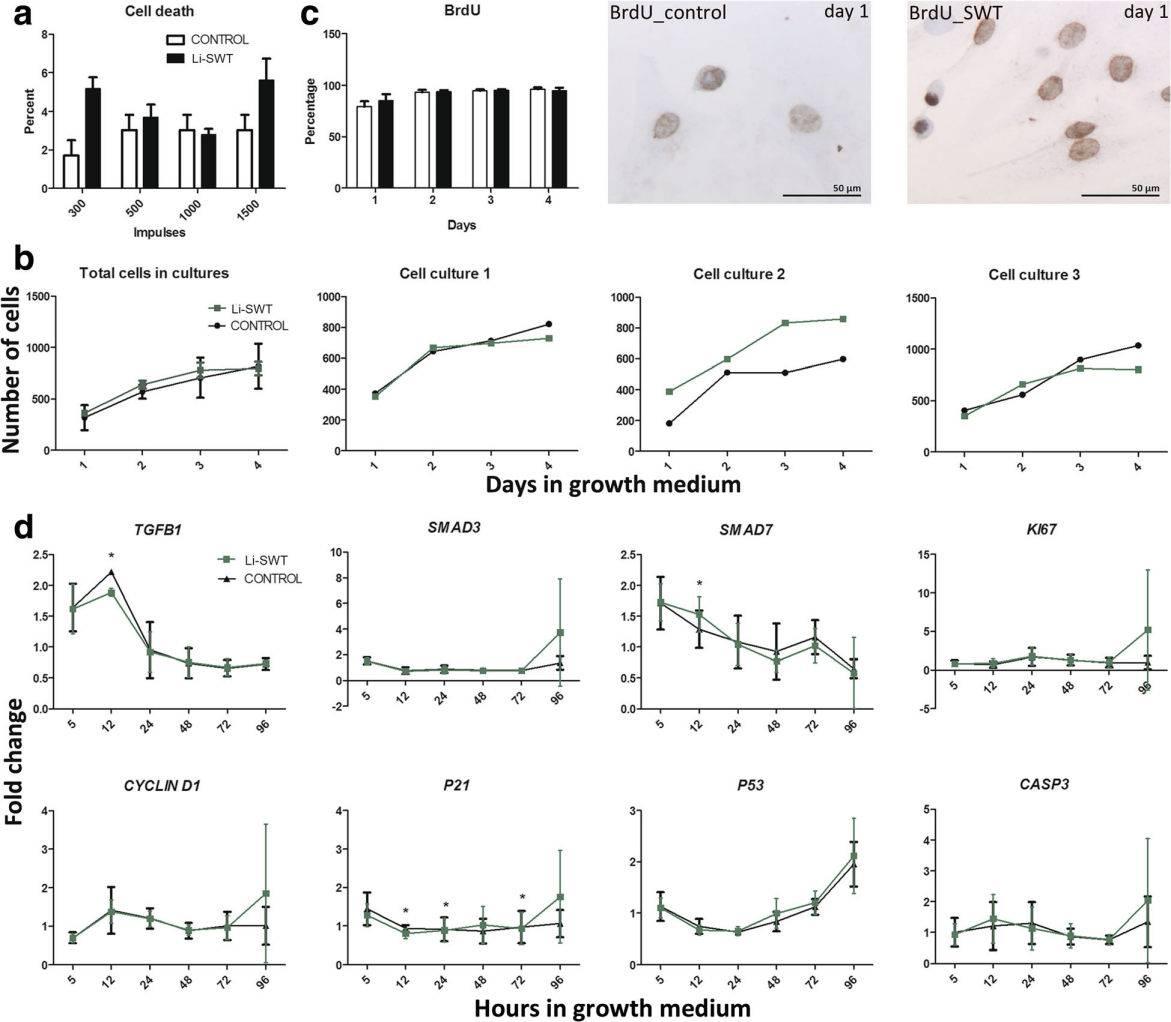
Quantifications of cell death, total cell counts, BrdU incorporation in cell cultures and gene expressions of *TGFB1, SMAD3, SMAD7, KI67, CYCLIN D1, P21, P53* and *CASP3* during myoblast proliferation. **a** Viability testing by tryphan blue at 300500, 1000 and 1500 impulses (0.1 mJ/mm^2^, 5hz) with 3 human cell cultures showed a small, but insignificant, increase in cell death after Li-SWT. **b** The myoblast proliferation was assessed in controls and after Li-SWT by detaching and counting the cells every day for 4 days. Variation between cell cultures was found, but no effect of Li-SWT was observed. **c** Proliferation was further investigated by BrdU incorporation followed by immunostainings, which revealed a 2% increase in proliferation on day 1 after Li-SWT. **d** Gene expression study during proliferation revealed that *TGFB1* expression was significant lower at 12 h after Li-SWT while *SMAD7* was higher expressed in Li-SWT compared to controls. *P21* was significant different at 12 h, 24 h and 72 h while *SMAD3, KI67, CYCKLIND1, P53 and CASP3* showed no difference between treatment groups.*n* = 3, * *p* < 0.05

Cells were treated with 300, 500, 1000 and 1500 impulses of Li-SWT and cell death subsequently quantified by tryphan blue uptake. Cell death was between 2.8–5.6% in the Li-SWT treated compared to 1.7–3.0% in controls, and thus we found no significant difference in cell death (Fig. 1a).

The effect of Li-SWT on myoblast proliferation was assessed by counting the number of cells on day 1, 2, 3 and 4 after treatment. The results showed some variation between the three cell cultures, but overall no significant difference between shockwave treated and controls was observed (Fig. 1b), nor did the BrdU incorporation studies demonstrate increased proliferation in treated cells compared to controls (Fig. 1c).

The expression of *TGFB1* during proliferation was significantly decreased at 12 h in the Li-SWT group, while *SMAD7* in this group at 12 h was significantly increased, yet there was no difference between the groups in *SMAD3* and *CASP3* expression (Fig. 1d). The cell cycle related genes *KI67*, *CYCLIND1* and *P53* showed no difference in expression (Fig. 1d), except *P21* was significantly different at 12 h, 24 h and 72 h. Thus, the data supports that Li-SWT exerted neither negative nor positive effects on cell survival and proliferation of myoblasts.

**Fig. 2:**
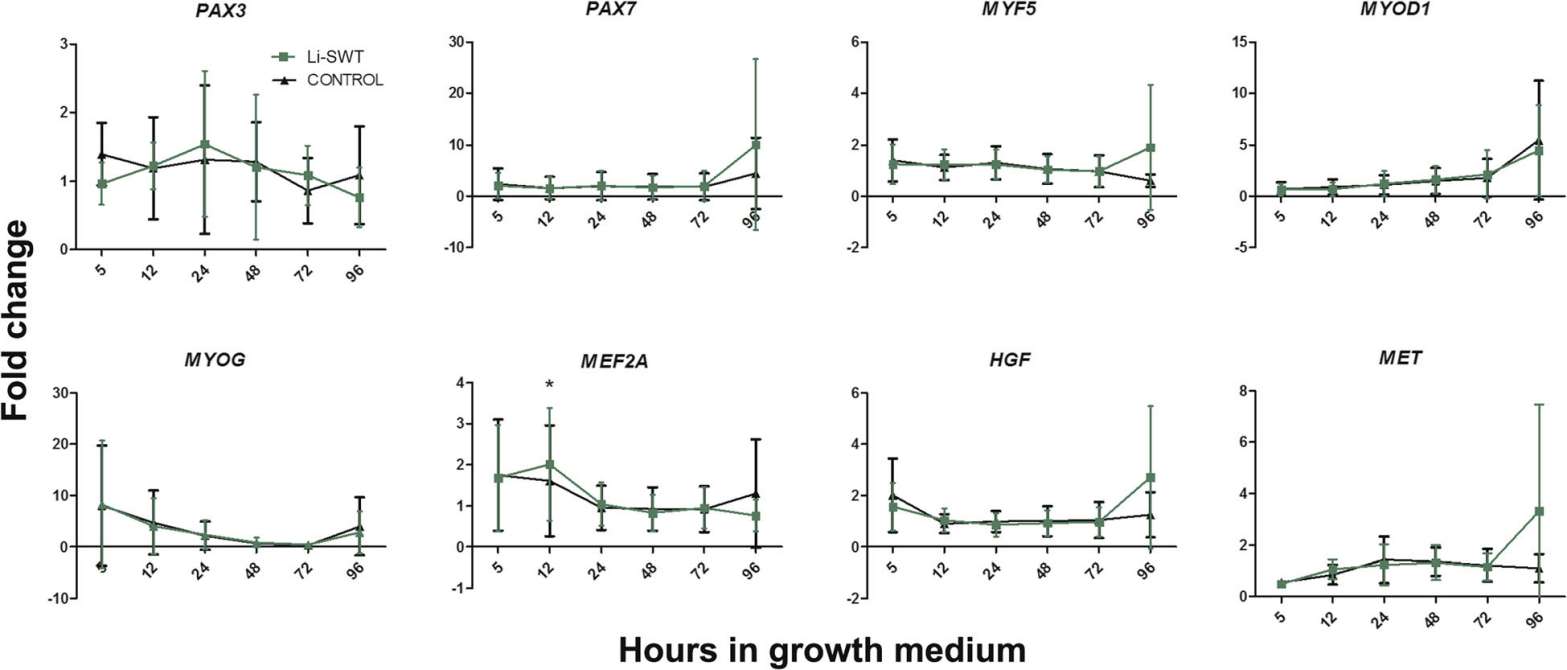
In vitro gene expression of *PAX3, PAX7, MYF5, MYOD1, MYOG, MEF2A, HGF* and *MET* during proliferation in control and shockwave treated myoblasts. *MEF2A* was significantly increased at 12 h after Li-SWT, while the expression of *PAX3, PAX7, MYF5, MYOD1, MYOG, HGF and MET* did not differ between treatment groups.n = 3, * p < 0.05

No difference was observed in the expression of the myogenic factors *PAX3*, *PAX7*, *MYF5*, *MYOD1*, *MYOG*, *MET*, and *HGF* genes during proliferation. However, *MEF2A*, a co-actor in myoblast differentiation [32], was significantly increased at 12 h in the Li-SWT group compared to control (Fig. 2).

### Li-SWT did not influence the migration ability in vitro

A scratch test performed 48 h after Li-SWT treatment with a duration of 18 h revealed no change in the ability of human Li-SWT myoblasts to migrate compared to controls. (*See* Additional file 2 *for images and detailed method*).

### Li-SWT did not alter myogenesis in vitro

**Fig. 3:**
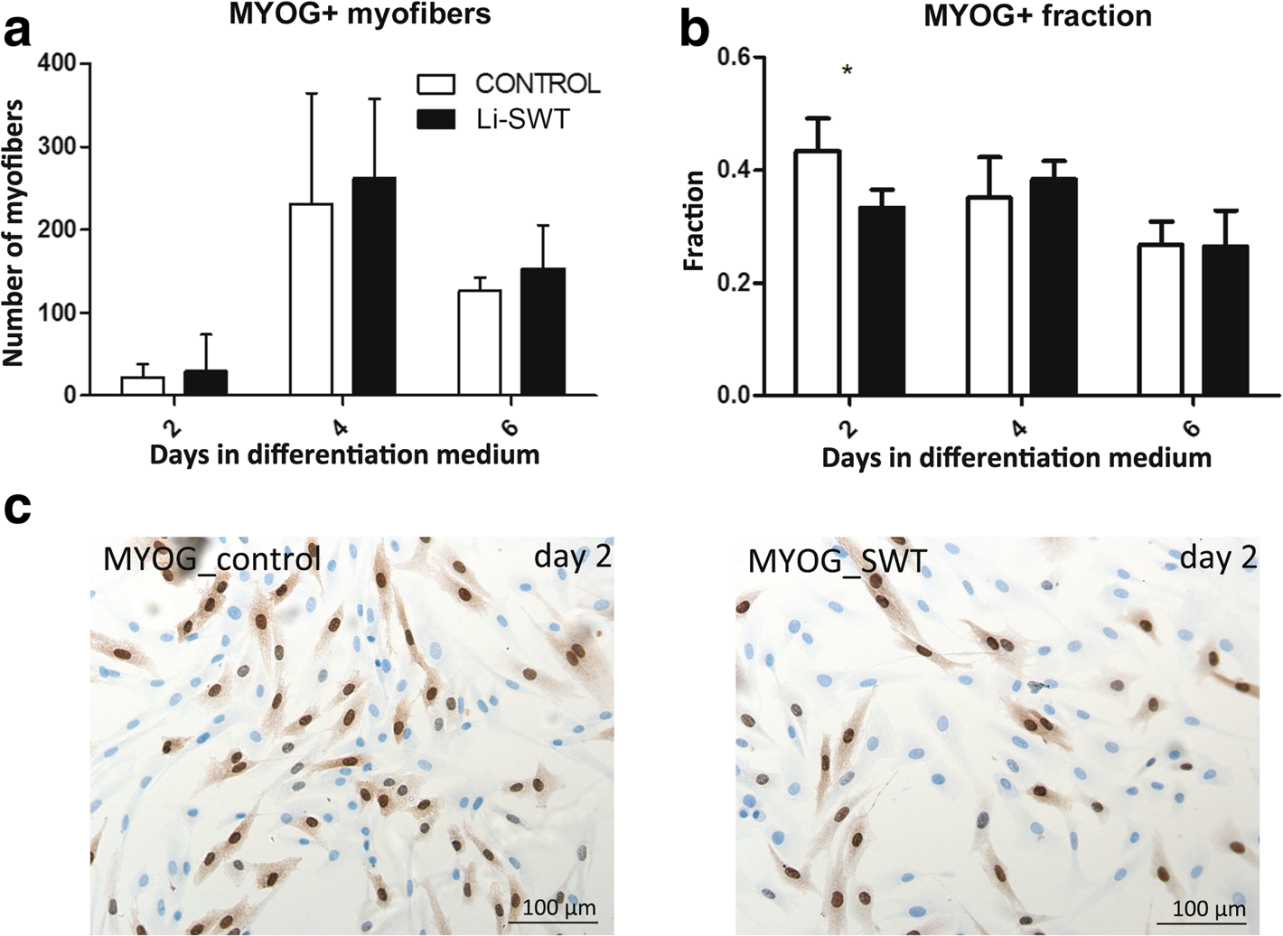
The number of MYOG positive myofibers and the fraction of MYOG positive cells during myoblast differentiation after Li-SWT. Myoblasts were treated with shockwaves and cultured in differentiation medium and harvested 2, 4 and 6 days after Li-SWT. **a** We found no difference in the number of myofibers (>2 nuclei) in control and Li-SWT myoblasts, thus cells had maintained their capacity to differentiate after Li-SWT. **b** The fraction of MYOG positive nuclei was significantly lower in Li-SWT cultures on day 2 during differentiation. However on day 4 and 6, the MYOG fractions were not changed by Li-SWT. **c** Immunohistochemical stainings for MYOG on day 2 after Li-SWT.*n* = 3, * *p* < 0.05

In order to determine if shockwaves affect the differentiation, myofiber (>2 nuclei) formation was quantified after culturing in differentiation medium for 2, 4 and 6 days. As an additional parameter the fraction of MYOG positive nuclei was estimated. Though no difference was seen in presence of myofibers (Fig. 3a), the fraction of MYOG+ cells was significantly lower on day 2 in the Li-SWT group (Fig. 3b). Although the number of MYOG+ cell thus seemed lower during induction of differentiation, the capacity for myofiber formation and the overall ability to differentiate was not impaired by Li-SWT.

### Decreased adhesion of explants after Li-SWT

**Fig. 4:**
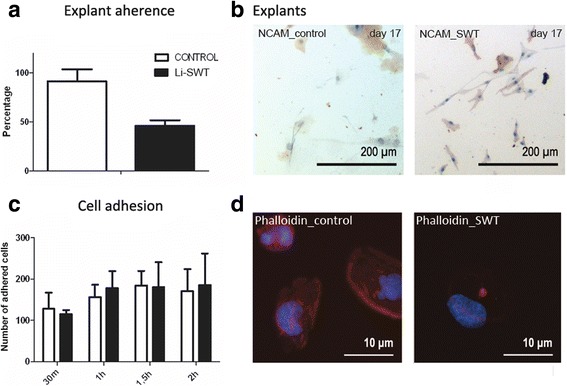
Quantifications of adherence of explants, outgrowth visualisations, cell adhesion and phalloidin staining of cells after shockwave treatment. **a** In explant cultures treated with 300 impulses of 0.1 mJ/mm^2^ 46% of the treated explants adhered compared to 92% in controls (*n* = 48), however (**b**) the number of cells grown out from the explants and their expression of NCAM did not differ. **c** The experiment was then performed with human myoblasts isolated by enzyme dissociation. The cells were cultured on coverslips and harvested every 30 min the first 2 h; no difference in adherence was observed. (**d**) However, when the harvested coverslips were stained for F-actin with phalloidin, the shockwave treated cells displayed a much lower expression of F-actin, indicating a delayed actin assembly. The shown images are from cells harvested 90 min after culture.n = 3, * p < 0.05

We found that 46% of the Li-SWT treated explants compared to 92% in controls (*n* = 48) adhered to the coverslips after 7 days (Fig. 4a). However, no difference was seen in the extent of outgrown cells or NCAM protein expression in the Li-SWT group compared to controls (Fig. 4b).

The adhesion kinetics of primary isolated myoblasts (isolated by enzyme dissociation) were then tested by treating the cells with Li-SWT followed by cell harvest every 30 min for the first 2 h after seeding. No reduced adherence following Li-SWT was seen in treated cells compared to controls (Fig. 4c.). However, the expression of F-actin, visualized by phalloidin staining, was reduced in Li-SWT treated cells, indicating a delayed formation of F-actin (Fig. 4d).

### In vivo

The effects of Li-SWT on skeletal muscle regeneration were studied in mouse muscle using a cardiotoxin-induced lesion that provides a complete regenerating microenvironment. The cardiotoxin lesion did not cover the entire TA muscle, hence the histological sections contained partly normal and partly regenerating area (Additional file 3 *)*.

### Expression of apoptosis related genes increased during early regeneration in shockwave treated animals

**Fig. 5:**
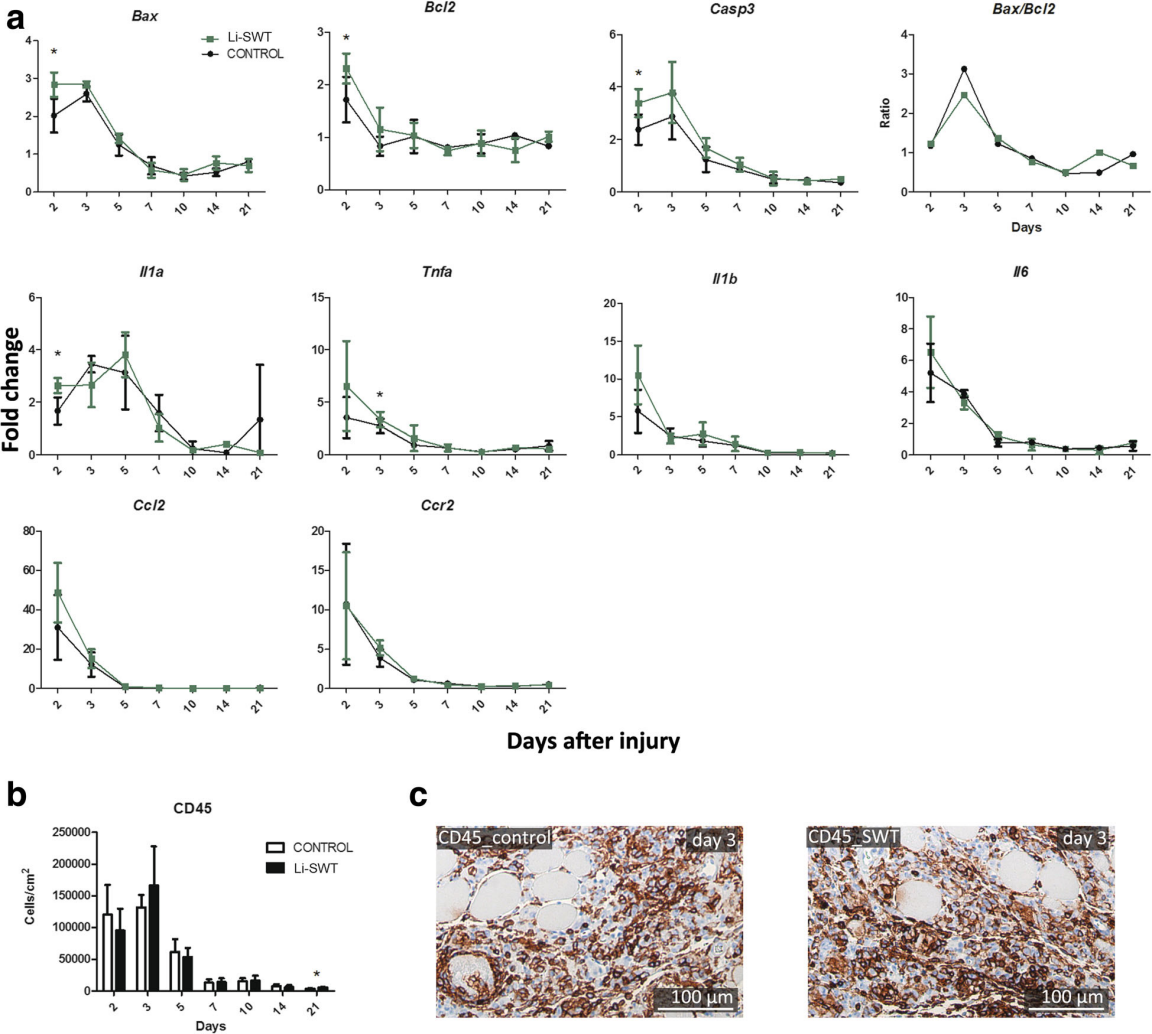
Expressions *of Bax, Bcl2, Casp3, Il1a, Il1b, Il6, Ccl2, Ccr2* and *Tnf*α and CD45+ cells during muscle regeneration in control and shockwave treated mice. **a** *Bax, Bcl2* and *Casp3* were highly expressed in the early phase of regeneration, and significantly different in the Li-SWT group on day 2, while the apoptotic potential, *Bax/Bcl2* seemed lower in the Li-SWT group on day 3. Similarly *Il1a and Tnf*α were significantly higher expressed in the early phase of regeneration. The expression of *Il1b, Il6, Ccl2 and Ccr2* showed similar expression pattern with highest levels from day 2–5 with no difference between the control and Li-SWT group. **b** Quantifications of CD45+ cells showed increased lymphocyte infiltration on day 2 and 3, with the only significant change in the number of CD45+ cells on day 21 in the SWT group. **c** Immunohistochemical stainings for CD45 on day 3. n = 3 in gene expressions and n = 4 in quantifications, * p < 0.05

Expression of the apoptosis related genes *Bax, Bcl2* and *Casp3* increased significantly on day 2 in shockwave treated legs compared to controls, indicating a stronger onset of apoptosis. The Bax/Bcl2 ratio, which determines the apoptotic potential [33], peaked at day 3 and decreased until day 10 for both groups, reflecting the muscle degeneration observed as part of the regeneration process [34, 35] (Fig. 5a).

### Small effect of Li-SWT on the early inflammatory response

The inflammatory response induced by the cardiotoxin lesion caused a significant increased *Il1a* expression on day 2, and *Tnfa* on day 3 in the Li-SWT legs, but no difference in the expression of *Il1b, Il6*, *Ccl2* and *Ccr2* was found between the groups*.* All genes were initially expressed at high levels and decreased towards day 10 with no further changes (Fig. 5a).

CD45 staining for inflammatory cells on the histological sections demonstrated a lesion-induced inflammatory cells on day 2 and 3, but no difference was observed between the groups except on day 21 where the infiltration was significantly higher in the Li-SWT legs (Fig. 5b, c).

Thus, a small increase in the early inflammatory signal may have occurred; however, the overall inflammatory response was not altered by the Li-SWT.

### Li-SWT caused upregulation of myogenic factors in vivo

**Fig. 6:**
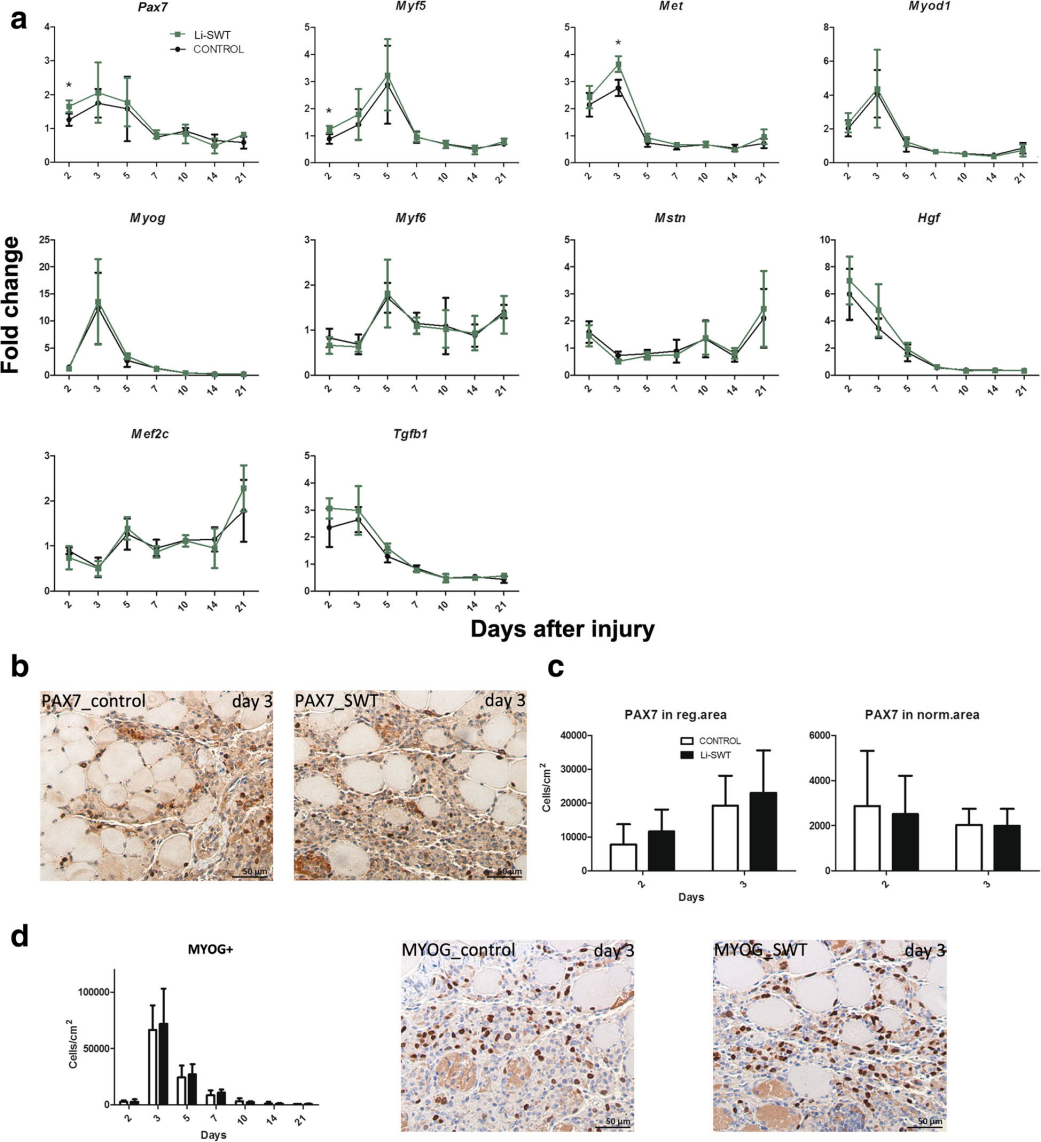
In vivo gene expression of *Pax7, Myf5, Myod1, Myog, Mstn, Myf6, Hgf, Met, Mef2c, Tgfb1* and protein expression of PAX7 and MYOG. **a** *Pax7, Myf5* were significantly increased on day 2 in the Li-SWT group, while *Met* was significantly increased on day 3 in the Li-SWT group. No difference was found between treatment groups in the expression of *Myod1, Myog, Myf6, Mstn, Hgf, Mef2c and Tgfb1.* **b** Immunohistochemical staining for PAX7 was made for Li-SWT and controls and the expression on day 3 is shown. **c** The PAX7 stainings were quantified, and the highest number of satellite cells (PAX7+) was found as expected in regenerating areas of the muscle, however no difference was observed in the number of PAX7+ cells in Li-SWT compared to control. **d** The number of MYOG+ cells peaked on day 3 but no difference was observed between the Li-SWT and control group. MYOG stainings are shown for day 3. n = 3 in gene expressions and n = 4 in quantifications, * p < 0.05

*Pax7, Myf5* and *Met* gene expressions were significantly increased on day 2 in the Li-SWT group. No difference was observed in the expression of *Myod1, Myog, Myf6, Mstn, Hgf, Mef2c* and *Tgfb1* (Fig. 6a).

Immunohistochemical stainings for the satellite cell marker PAX7 was made (Fig. 6b) and the stainings were quantified on day 2 and 3 (Fig. 6c). As expected a higher density of PAX7 positive cells was observed in the regenerating areas, however we found no difference in the number of PAX7+ cells between the treated and control legs. Likewise, a large number of MYOG+ cells were observed in the regenerating areas on day 2 and 3, however no difference between Li-SWT and controls was found (Fig. 6d).

Thus gene expression studies indicate a Li-SWT induced early SC activation though the number of cells expressing the myogenic markers PAX7 and MYOG was unaltered.

### Li-SWT increased the angiogenic gene expression in vivo

**Fig. 7:**
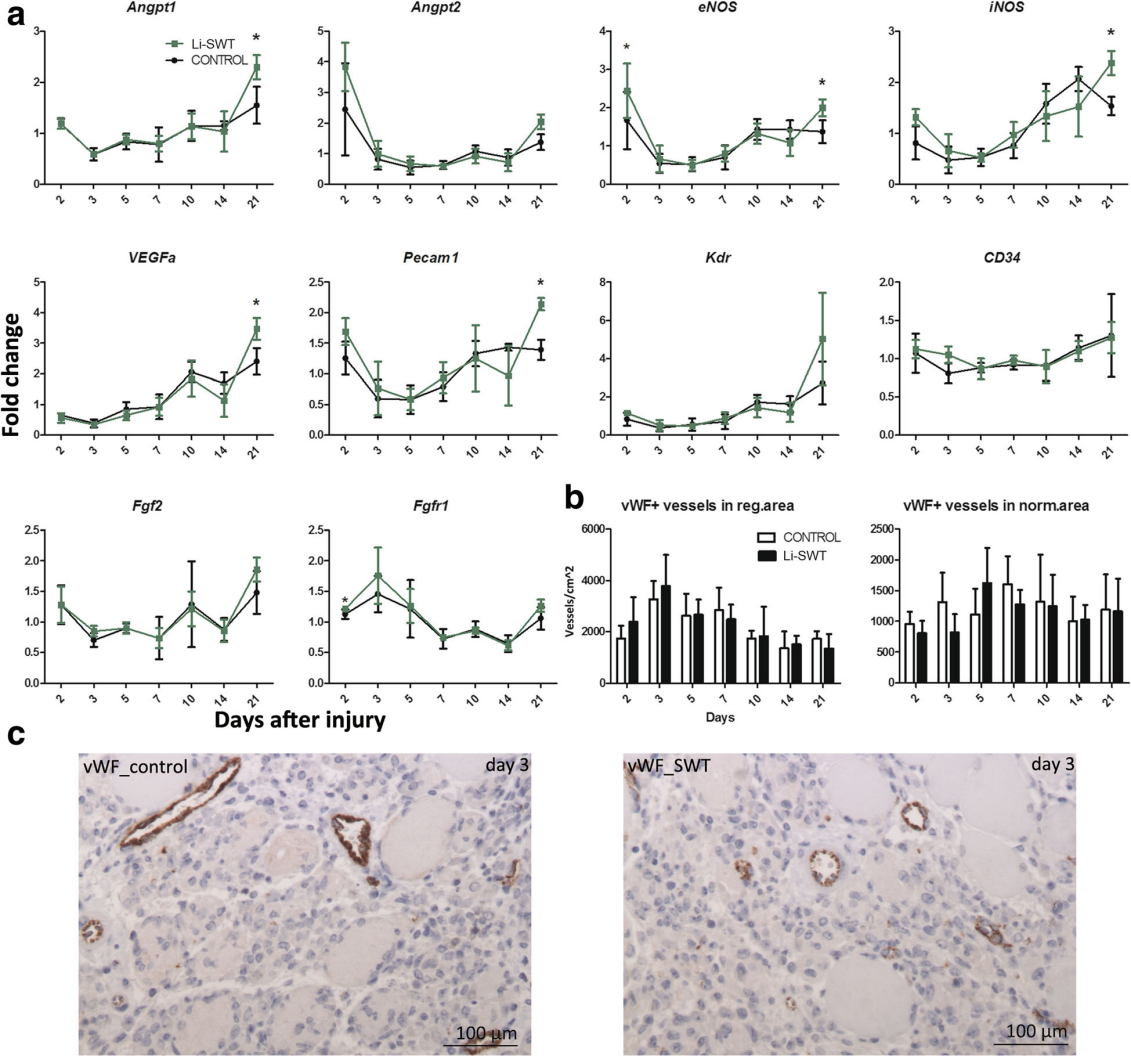
Expression of *Angpt1, Angpt2, eNOS, iNOS, VEGFa, Pecam1, Kdr, CD34, Fgf2* and *Fgfr1* and vessel density during muscle regeneration in control and Li-SWT mice. **a** *Angpt2*, *eNOS* and *iNOS* were high expressed on day 2 in Li-SWT, with *eNOS* being significantly higher expressed in the Li-SWT group. *Angpt1, eNOS, iNOS, VEGFa* and *Pecam1* were significant up regulated on day 21 in the Li-SWT group. Thus Li-SWT induced an early and late angiogenic signal. No effect of Li-SWT was found in expression of *Kdr, CD34, Fgf2,* but *Fgfr1* was significantly increased in the Li-SWT group on day 2*.* **b** and (**c**) Blood vessels were visualized by immunostainings for vWF and the vessel density (number of vessels/cm^2^) peaked on day 3 in regenerating areas before decreasing again. No effect of Li-SWT on vessel density was found in either regenerating- or normal muscle areas. **c** Immunostainings for vWF are shown for day 3 in Li-SWT and controls.n = 3 in gene expressions and n = 4 in quantifications, * p < 0.05

The gene expression of *Angpt1, Angpt2, eNos and iNos* were high on day 2 in the Li-SWT group. *eNos* was significantly increased in the Li-SWT group on day 2 and *iNos* and *Angpt2* showed the same tendency (Fig. 7a). This could indicate that Li-SWT supports early angiogenesis during muscle repair. On day 21 *Angpt1, eNos, iNos, Vegfa*, and *Pecam1* were significantly higher expressed in Li-SWT treated legs, with *Angpt*2 and *Vegfr2* having the same tendency (Fig. 7a), indicating a late effect, too, of Li-SWT, corresponding to previous studies [21, 36].

*CD34* gene expression increased slightly in both groups from day 10, but there was no difference between treated and control legs. Furthermore there was no difference in the *Fgf2* expression, a well-known co-player in angiogenesis [37], while the expression of *Fgfr1* was significantly increased on day 2 in Li-SWT treated compared to control (Fig. 7a).

Quantitation of vessel density by immunohistochemial staining with vWF demonstrated increased vessel density in the regeneration area compared to the unaffected area of the muscle, but there was no difference between treatment groups (Fig. 7b,c). Though no difference in vessel density was found, the gene expressions indicate an enhanced angiogenic signal caused by Li-SWT.

## Discussion

Our study is the first to present the effect of Li-SWT on human myoblast cultures and mouse skeletal muscle undergoing regeneration after acute injury. The beneficial effect of Li-SWT treatment on microcirculation has been documented in various tissues [21, 25, 38, 39] but not much is known about how Li-SWT affects the skeletal muscle specific stem cells.

### Li-SWT exerted no harmful effect on human myoblasts

The viability of myoblasts was tested, and within the range of 300 to 1500 impulses the energy application was well tolerated. For further in vitro studies a dose of 300 impulses of 0.1 mJ/mm^2^ was used. Similar in vitro studies have used impulses ranging from 250 to 1000 depending on cell type [17, 19, 40]. When myoblast cultures were treated with Li-SWT we found a very low level of cell death, comparable to studies conducted on osteoblasts and fibroblasts [40]. We also found that the proliferation potential of myoblasts and their ability to migrate and differentiate was unaffected by the Li-SWT with the applied dose. Thus Li-SWT was not harmful to myoblasts.

### Li-SWT altered expression of *TGFβ1, SMAD7 and MEF2A* during in vitro proliferation

Although Li-SWT had no effect on the number of proliferating cells, we found that the expression of *TGFβ1* was down regulated while *SMAD7, P21* and *MEF2A* were significantly up regulated in the Li-SWT treated cell cultures during proliferation. Thus, the *TGFβ1* signaling pathway seemed to be sensitive for shockwave treatment, however no conclusion on the overall influence of the pathway can be drawn from the present gene expression data. *MEF2A* is crucial for myoblast differentiation [32], and interestingly, it was significantly up regulated already 12 h after treatment. In C2C12 *MEF2A* is increased after mechanical stress in C2C12 myoblasts [41], thus the Li-SWT induced shear stress may have led to the up regulation of MEF2A in the human myoblasts.

### Myoblast stress tolerance

It is likely that various cell types can withstand different amount of stress. Tenocytes are often treated with 1000 impulses [16, 17], while cells of endothelial origin, which are sensitive to flow changes (shear stress) [42], tend to be treated with 300 impulses [19, 43]. Myoblasts may have a stress tolerance similar to tenocytes given their similar niche and ability to sustain continues stretch, which could explain the lack of effects of Li-SWT on proliferation and differentiation observed in this study.

### Decreased adhesion in explants and cell cultures after Li-SWT

Stress mechanotransduction can affect the cytoskeleton and focal adhesion proteins, leading to loss of adhesion [44]. Likewise, shockwave treatment has been reported to result in cell detachment in monolayers of cardiac cell cultures [20] and renal carcinoma cell line [45],. However, increased adhesion has been reported in suspended osteoblasts treated with Li-SWT [46]. Thus, the effect of Li-SWT seems to depend on the method of application and cell type.

The decreased attachment of Li-SW treated explants observed in our study could be due to an initial effect of Li-SWT. Detached renal carcinoma cells showed actin depolymerisation and altered actin filament organisation [45]. Likewise, we observed a decreased assembly of F-actin after Li-SWT. Although we did not observe altered attachment properties, this indicates an effect of Li-SWT on myoblast cytoskeleton.

### Li-SWT affected the muscle regeneration in mice

In our study Li-SWT significantly increased apoptotic, pro-inflammatory and myogenic factors in the early phase of regeneration, probably a result of shockwave-induced stress in an already necrotic/apoptotic environment [33].

Although the *Bax/Bcl-2* ratio indicated that apoptotic activity was reduced on day 3 in the treated legs, the significant expression of *Bax and Casp3* on the previous day implied a Li-SWT-induced pro-apoptotic gene expression. Other studies have reported decreased necrosis and decreased amount of cells undergoing apoptosis in ischaemic skin flaps after Li-SWT [22, 47], however, an ischaemic skin flap is different from regenerating muscle in an otherwise healthy mouse, and the response upon injury depends on the type and the extend of the injury, for instance degeneration and regeneration progress faster after myotoxic injury than after ischaemic lesions [35, 48].

In the inflammatory markers *Il1a* and *Tnfa* was significantly increased in early regeneration and *Il1b and Il6* showed the same tendency. However, quantifications of leucocytes did not reveal increased infiltration nor was there any increase in the *Ccl2* expression, indicating similar monocyte/macrophage infiltration [49]. In contrast a recent study reported significantly decreased expression of pro-inflammatory genes and leukocyte infiltration in burn injuries after Li-SWT [50] and another study reported decreased leukocyte rolling and transmigration of endothelium after Li-SWT [25].

Though Li-SWT did not augment the number of PAX7+ satellite cells, an increased expression of *Pax7*, *Myf5* and *Met* on day 2, indicate increased SC induction. Thus Li-SWT may have induced the regenerative response in the acute muscle injury model, in accordance with reported observations on the effect of pro-inflammatory cytokines, HGF release and shear stress [34, 51–53]. The effect might prove even more beneficial in a chronic injury model, where the background is suboptimal repair.

### Increased angiogenic response in mice

We found in the regenerating muscle an increased expression of *eNOS* on day 2 after Li-SWT and the same tendency was observed in *iNOS* and *Angpt2*, indicating an early angiogenic reponse. Furthermore*, Angpt1, eNOS, iNOS, Vegfa*, and *Pecam1* gene expressions increased from day 14 to 21 in the Li-SWT group,

The increased *eNOS* and *iNOS* expression was most likely caused by shockwave-induced increase in intracellular calcium, which activates *eNOS* and *iNOS* [54]. Further, iNOS is induced by pro-inflammatory cytokines like TNF-a and IL1b [55]. In addition, shear stress is known to contribute to increased eNOS expression [56] and increased release of angpt2 from endothelial cells [57], which could potentially lead to increased angiogenesis.

Despite Li-SWT induction of pro-angiogenic genes the vessel density was unaffected, which is in contrast to recent studies reporting increased vessel density after treatment of ischaemic muscle or skin [23, 24, 26]. Thus, the injury model, might be crucial for SW-induced acceleration of regeneration. The mice used in the present study were healthy and thus fully capable of regenerating a cardiotoxin lesion without Li-SWT. To be demonstrable a Li-SWT induced angiogenic response may need to be studied in a disease model where the regeneration capacity as a starting point is insufficient.

In a clinical perspective the increased pro-angiogenic gene expression and the early stimulation of satellite cells induced by Li-SWT might be beneficial in regenerating skeletal muscle injuries. Especially the field of skeletal muscle tissue engineering with the use of scaffolds where activation of myoblasts and proper vascularization are limiting factors for survival and integration of transplanted tissue, treatment with Li-SWT might enhance the engraftment and regeneration process. Likewise support of regeneration in muscle lesions in areas with insufficient blood supply might be a target for Li-SWT.

Shockwave treatment is commonly applied to tissues covered by or adjacent to muscle and thus skeletal muscle is included in the treatment area. It is therefore an important information that muscle apparently is not injured by shockwave treatment.

Many articles have reported the effect of Li-SWT, but this study describes a large number of genes affected by Li-SWT, which to our knowledge has never been described before.

## Conclusions

Our studies have shown, that Li-SWT is well tolerated and that when applied to muscle stem cells in vitro and muscle in vivo with a total energy of 0.981 J and 1.635 J respectively cellular changes were induced that could promote muscle regeneration.

## Additional files

Additional file 1: Numerical data (fold changes) from the in vitro and in vivo gene expression studies. The table shows the numerical data (fold changes) from the in vitro gene expression studies (Fig. 1 and Fig. 2) and the numerical data from the in vivo gene expression studies (Fig. 5, Fig. 6 and Fig. 7). (XLSX 58 kb)

Additional file 2: Migrating ability after Li-SWT. A scratch test performed 48 h after Li-SWT treatment with a duration of 18 h revealed no change in the ability of human Li-SWT myoblasts to migrate compared to controls. (TIFF 1198 kb)

Additional file 3: HE stainings of the regeneration process after CTX injury with and without Li-SWT on paired hindlimbs. The extend of injury caused by cardiotoxin varied between hindlimbs, but overall no adverse affects of Li-SWT was found. White arrows point to normal muscle areas, while black arrows point to regenerative areas. In the upper right corner of each picture the regenerative area is upscaled. Scalebars represents 100 μm. (TIFF 8648 kb)

## Abbreviations

Li-SWT: Low intensity shockwave therapy
SC: Satellite cells, BrdU: Bromodeoxyuridine
