# Supplementary figures and images for: The effect of low intensity shockwave treatment (Li-SWT) on human myoblasts and mouse skeletal muscle
Source: BMC Musculoskelet Disord. 2017 Dec 29;18:557. doi: 10.1186/s12891-017-1879-4 (PMC5747105; doi:10.1186/s12891-017-1879-4)

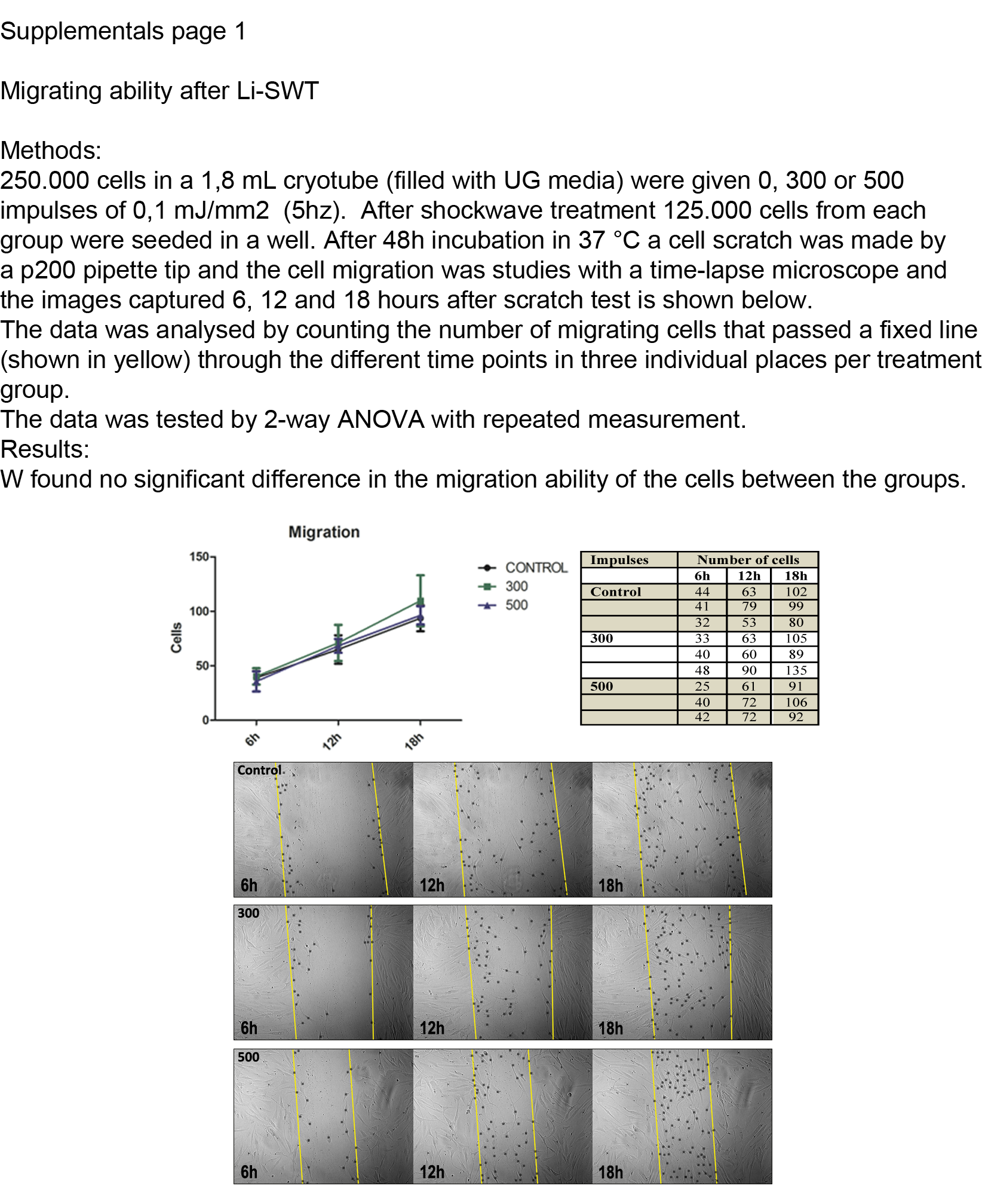

Supplement: Supplementary file 2 — Migrating ability after Li-SWT. A scratch test performed 48 h after Li-SWT treatment with a duration of 18 h revealed no change in the ability of human Li-SWT myoblasts to migrate compared to controls. (TIFF 1198 kb) [file 12891_2017_1879_MOESM2_ESM.tif]

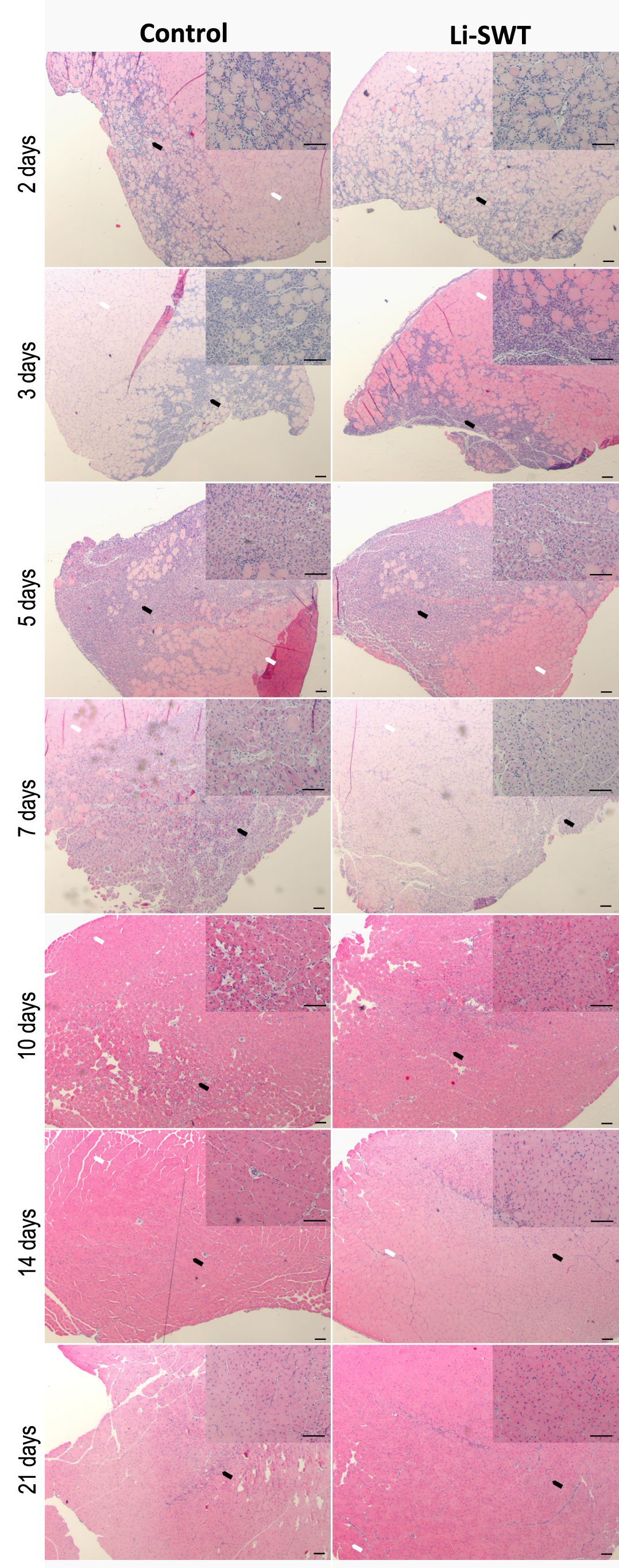

Supplement: Supplementary file 3 — HE stainings of the regeneration process after CTX injury with and without Li-SWT on paired hindlimbs. The extend of injury caused by cardiotoxin varied between hindlimbs, but overall no adverse affects of Li-SWT was found. White arrows point to normal muscle areas, while black arrows point to regenerative areas. In the upper right corner of each picture the regenerative area is upscaled. Scalebars represents 100 μm. (TIFF 8648 kb) [file 12891_2017_1879_MOESM3_ESM.tif]
